# Editorial

**Published:** 1842-02-26

**Authors:** 


### THE MEDICAL EXAMINER.

PHILADELPHIA, FEBRUARY 26, 1842.

*Dr. Pancoast's case of amputation at the knee-joint.*—On Saturday last we enjoyed the opportunity of again examining the stump in this case, the operation upon which was reported in the last number of the Examiner for 1841. By the politeness of the operator, we have been favoured, also, with the perusal of the notes of the case during the earlier part of the after treatment, as preserved Dr. J. Livingston Ludlow, House Surgeon in charge of the ward. The only accident of any importance following the operation was twitching and spasm of the psoas and ilieus muscles, bringing the thigh to a right angle with the body, and requiring that the limb should be bound down to the bed, over the pillow, by means of rollers and straps—an object which was only accomplished by the exercise of the "whole force" of Dr. Ludlow. Under the use of fifteen drops of laudanum, frequently repeated, the spasms subsided in a few hours. The spasms were confined to the day of the operation. About a drachm of synovia was discharged at the first dressing, and this amount was at no time exceeded. The patient has not lost sleep at any time since the operation. She states that she has suffered no pain except from the pressure of the dressings in compressing the flaps. The clinical remarks of Dr. Pancoast inform us that a portion of articular cartilage continued visible so long as to leave the changes which it underwent subject to inspection until its removal. The first deep-seated granulations appeared to spring from the lateral parts of the joint, and from the neighbourhood of the attachments of the crucial ligaments. No change was visible on the cartilaginous surface until the whole cartilage assumed the appearance of having been macerated for a long time, fell away in flakes, from time to time, and laid bare the osseous surface of the condyles, which was obviously formed of a very thin, smooth lamen of osseous matter, covering the cancelli. Beneath this pellicle the red and enlarging vessels were distinctly obvious, and from it, or apparently through it, finally arose the healthy granulations which have now completed the union of the flaps with the bone.

On inspection, we found but a small surface still suppurating in the middle of the eschar. It is entirely superficial. The whole eschar occupies a small space, chiefly near the ham, and efficiently protected from pressure in using a wooden leg. The flap from the front of the leg, which was curtailed so greatly by the extent of the disease, now covers fully one-third of the osseous stump; but the greatest pressure will ultimately bear on the lateral flaps. The patella is drawn up a short distance above the condyles, and is perfectly moveable,—the bursa above the joint evidently remaining unaltered. There is no sign of the remains of a synovial cavity in any part of the stump. The mass of matter covering the bone remains, as yet, thick and elastic, and the termination of the case may be termed exceedingly happy—the only remaining question of importance being the capacity of the integuments of the lateral flaps to support the weight of the body when the edges of the condyles shall come to be supported on a wooden leg.

